# The Value of Disinfectants

**Published:** 1921-11

**Authors:** 


*To the Editor of* THE HOSPITAL AND HEALTH REVIEW.

### The Value of Disinfectants.

*To the Editor of* THE HOSPITAL AND HEALTH REVIEW.

SIR,—There seems to be some argument in favour of what one of your correspondents said last month about chemical disinfectants. They are not cheap, and if they are used for the purpose of attempting to sterilise drains and sewers, well, as he says, it is a waste of money. You might just as well try to sterilise the intestinal canal. It can't

**CORRESPONDENCE—(*cont.*).**

be done. But disinfectants are useful, chiefly, I think, because people will scrub out rooms and infected places if they are given some disinfectant to use, whereas they would not take the trouble if they had only soap and water. As for the use of sulphur and formalin, they may not kill very many disease germs, but they make such a beastly smell in the house that the people have to open the doors and windows to let in the fresh air, which, together with the sunlight, is, of course, a fine and cheap disinfectant.—Yours truly, ANOTHER M.O.H.

